# A Bright, Nontoxic, and Non-aggregating red Fluorescent Protein for Long-Term Labeling of Fine Structures in Neurons

**DOI:** 10.3389/fcell.2022.893468

**Published:** 2022-06-29

**Authors:** Lin Ning, Yang Geng, Matthew Lovett-Barron, Xiaoman Niu, Mengying Deng, Liang Wang, Niloufar Ataie, Alex Sens, Ho-Leung Ng, Shoudeng Chen, Karl Deisseroth, Michael Z. Lin, Jun Chu

**Affiliations:** ^1^ Department of Neurobiology, Stanford University, Stanford, CA, United States; ^2^ Interdisciplinary Research Center on Biology and Chemistry, Shanghai Institute of Organic Chemistry, Chinese Academy of Sciences, China University of Chinese Academy of Sciences, Beijing, China; ^3^ Department of Bioengineering, Stanford University, Stanford, CA, United States; ^4^ Guangdong Provincial Key Laboratory of Biomedical Optical Imaging Technology and Center for Biomedical Optics and Molecular Imaging, and CAS Key Laboratory of Health Informatics, Shenzhen Institute of Advanced Technology, Chinese Academy of Sciences, Shenzhen, China; ^5^ Department of Biochemistry and Molecular Biophysics, Kansas State University, Manhattan, KS, United States; ^6^ Guangdong Provincial Key Laboratory of Biomedical Imaging, The Fifth Affiliated Hospital, Sun Yat-sen University, Zhuhai, China

**Keywords:** red fluorescent protein, RFP, crimson, non-aggregating, long-term, label, neuron

## Abstract

Red fluorescent proteins are useful as morphological markers in neurons, often complementing green fluorescent protein-based probes of neuronal activity. However, commonly used red fluorescent proteins show aggregation and toxicity in neurons or are dim. We report the engineering of a bright red fluorescent protein, Crimson, that enables long-term morphological labeling of neurons without aggregation or toxicity. Crimson is similar to mCherry and mKate2 in fluorescence spectra but is 100 and 28% greater in molecular brightness, respectively. We used a membrane-localized Crimson-CAAX to label thin neurites, dendritic spines and filopodia, enhancing detection of these small structures compared to cytosolic markers.

## Introduction

Fluorescent proteins (FPs) are valuable tools for visualizing cellular processes in living cells. After the cloning of *Aequoria victoria* green fluorescent protein (GFP), a variety of FPs with different wavelengths have been discovered in marine organisms (Day and Davidson, 2009). Red fluorescent proteins (RFPs) (emission peak in the 585–620 nm range) (Cranfill et al., 2016), including commonly used mCherry (Shaner et al., 2004), are particularly useful for 2-color imaging in conjunction with GFP or GFP-based indicators. Even for single-wavelength experiments, RFPs have advantages over GFPs in that their longer excitation wavelengths generate less phototoxicity and autofluorescence, and their longer emission wavelengths are scattered less severely *in vivo*. A variety of RFPs have been developed to improve brightness, maturation, and monomericity, while covering emission wavelengths from the red to the far-red (Shen et al., 2015; Ng and Lin, 2016). Some of these RFPs are even brighter than GFP and perform well when expressed alone or fused to subcellular tags in standard proliferating cell types (Cranfill et al., 2016).

One surprisingly uncommon use of RFPs, however, is in membrane labeling of mammalian neurons. In neurons, synapses are located on dendritic spines, mushroom-shaped membrane protrusions less than 1 μm^3^ in volume on which synapses form. Spines evolve from filopodia during neuronal differentiation, undergo morphological changes in response to neurotransmitter activation and during learning (Holtmaat et al., 2009; Xu et al., 2009), and exhibit abnormalities in number or morphology in several neurological diseases, such as the retention of filopodia-like morphology in Fragile X syndrome (Govek et al., 2004; Woolfrey et al., 2009; Sala and Segal, 2014). Thus, accurate visualization of filopodia spine number and morphology in living neurons over long experimental time-courses would be highly beneficial to the study of synaptic differentiation or pathogenesis. Membrane-targeting of GFP has been shown to improve detection of small neuronal structures (Craske et al., 2005; Han et al., 2011; Zhou et al., 2012; De Rubeis et al., 2013; Michaelsen-Preusse et al., 2016). However, due to the lower phototoxicity and autofluorescence at their excitation wavelengths, membrane-targeted RFPs would be useful as well. We and others have used mCherry-CAAX to label the neuronal membrane in transient transfection experiments (St-Pierre et al., 2014; Feng et al., 2019), but whether alternative RFPs may be brighter or exhibit less toxicity has not been explored.

In this study, we develop a bright, non-toxic RFP named Crimson with excitation and emission spectra similar to mCherry (Shaner et al., 2004). Crimson shows comparable maturation speed and higher molecular brightness compared to mCherry. Comparing the brightness and toxicity of Crimson to two RFPs [FusionRed (Shemiakina et al., 2012) and mCherry] and one far-red FP [mKate2 (Pletnev et al., 2008)] with excitation peak ranging from 580 to 590 nm, we found Crimson was both brightest and least toxic in both cytosolic and membrane-targeted forms. Moreover, Crimson did not exhibit lysosomal accumulations, as was observed in mCherry (Laviv et al., 2016) and mCherry-derived mScarlet (Fenno et al., 2020). Finally, we found that Crimson-CAAX enhanced detection of filopodia and spines compared to cytosolic fluorescent proteins.

## Materials and Methods

Mutagenesis and screening of libraries. Crimson is engineered from mNeptune2 by rational mutagenesis. Mutations at specific residues were introduced by overlap-extension PCR. All PCR products were cut and ligated into a constitutive bacterial expression vector pNCS (Allele Biotech). To easily screen bright Crimson variants, Clover-Crimson tandem fusions with high intramolecular FRET efficiency were constructed. Chemically competent *Escherichia coli* strain XL-10 Gold (Invitrogen) were transformed and grown overnight on LB/agar at 34°C and maintained thereafter at room temperature for ∼6 h. For each round of mutagenesis, a number of colonies approximately tenfold higher than the expected library diversity were screened to ensure full coverage. Agar plates were screened for transmitted color by eye and for fluorescence in a BlueView Transilluminator (Vernier) with 400- to 500-nm excitation light and a yellow acrylic long-pass filter. Bacterial colonies of interest were patched on LB/agar plates and incubated overnight at 34°C. Bacteria were resuspended in PBS or lysates were extracted with B-PER II (Pierce), and FRET spectra were obtained on a Safire II plate reader (Tecan). DNA sequences of all constructs are available upon request.

Characterization of Crimson *in vitro*. Fluorescent proteins with polyhistidine tags were expressed from pNCS vectors in XL-10 gold bacterial cells, purified with cobalt-chelating affinity chromatography (Pierce) and desalted into phosphate-buffered saline (PBS) pH 7.2 using gel filtration columns (Bio-Rad). Excitation spectra and emission spectra were measured with an Infinite M1000 fluorometer (Tecan). Extinction coefficients were calculated using the base-denaturation method (Shaner et al., 2004). Quantum yields were determined using mKate2 as a standard (QY = 0.40). pH titrations were performed using a series of pH buffers ranging from 2 to 10.5 (Citrate-Tris-Glycine buffer, 50 mM each. The desired pH was achieved by adding 2 M sodium hydroxide or 2 M hydrochloric acid). *In vitro* photobleaching measurements were performed in PBS droplets under mineral oil on an IX83 inverted microscope with a 40 × /1.25-numerical aperture (NA) oil-immersion objective, a 100-W metal halide lamp (Olympus) at 100% neutral density, a 580/15-nm excitation filter (Omega), and an optiMOS Scientific CMOS camera (Qimaging) controlled by Micro-Manager software. Images were acquired every 1 s under continuous illumination. Times were scaled to produce photon output rates of 1,000 per molecule per s as previously described (Shaner et al., 2004). Maturation experiments were performed by measuring change in fluorescence following exposure of Crimson-expressing *E. coli* grown in deoxygenated media to normal atmosphere. Size exclusion chromatography (SEC) was performed on a LC-20A (SHIMADZU) high-pressure liquid chromatography (HPLC) system with a Superdex 200 10/300 GL column (GE Bioscience). 200 μL of each fluorescent protein at concentration 10 μM or 333 μM (∼10 mg/mL) were loaded. The column was operated at the flow rate of 0.5 mL/min with 50 mM PBS (pH 7) as the mobile phase. *In vitro* photobleaching measurements were performed in aqueous droplets of purified proteins in mineral oil using an Olympus IX83 inverted microscope with a 40 × /1.25-numerical aperture (NA) silicone oil-immersion objective (Olympus), an X-cite 120-W metal halide lamp (Lumen Dynamics) and a 568/20 nm excitation filter (Omega). The illumination power at the objective was 20 mW. Images were taken every 1 s under continuous illumination. Times were adjusted to produce photon output rates of 1,000 per molecule per second as described previously.

Characterization of RhoA FRET sensors with green/red FRET pairs. To construct RhoA-GR, three PCR fragments encoding truncated dClover2 (aa 1–217, Clover-N149Y/G160S), RhoA sensing domains from Raichu-RhoA FRET sensors and full-length RFP (mRuby3 or Crimson) were ligated into modified pcDNA3.1 vector using In-Fusion kit. HeLa cells were maintained in high glucose Dulbecco’s Modified Eagle Medium (DMEMClone) supplemented with 10% FBS (Invitrogen) and 1% penicillin-streptomycin (HyClone) at 37°C in air with 5% CO_2_. Cells were transfected at 80–90% confluency with Lipofectamine 2000 (Invitrogen) in 35 mm dishes. Transfections were carried out according to manufacturer’s instructions.

To determine the green/red emission ratio change of RhoA-GR with dClover2-mRuby3 or dClover2-Crismon, transfected cells expressing RhoA-GR were trypsinized and transferred to 96-well glass-bottom microplate (Cellvis) after 48 h transfection. Cells were allowed to settle down to the bottom of a microplate for 10 min at room temperature. Fluorescence spectra on transfected cells were obtained on an Infinite M1000 PRO (TECAN) fluorometer using 450-nm excitation light with 10-nm bandwidth. Emission was collected from 470 to 750 nm in 2-nm steps with 10-nm bandwidth. The red/green emission ratio was calculated from integrated red emission (mRuby3: 560–750 nm, Crimson: 580–750 nm) divided by integrated green emission (500–550 nm).

Constructs for neuron imaging. For pcDNA3.1-RFP cyto plasmids, each RFP expression cassette was amplified by PCR from parent template, and then cloned into pcDNA3.1 empty vector by infusion cloning method. For pcDNA3.1-RFP-CAAX plasmids, CAAX sequence was synthesized as part of In-Fusion cloning primer and subsequent cloning steps were performed as described in In-Fusion kit (Takara Bio, # 639650). In order to have the expression of two-color FPs in neurons at 1:1 ratio, we utilized a bi-directional plasmid, which was created in the lab earlier. In this special expression construct, A CMV enhancer region is flanked bi-directionally by a miniCMV promoter and a CAGGs promoter; either direction can drive the expression of one target protein independently. Crimson-CAAX and mTurquoise2 were then cloned into this plasmid by In-Fusion cloning method with mTurquoise2 driven by the β-actin promoter and RFP-CAAX driven by the mini-β-actin promoter. For zebrafish expression, we fused mTurquoise2 with Crimson-CAAX with a P2A peptide in between, and then subcloned the fused sequence into a Tol2 plasmid.

Neuron cell culture. All cell culture reagents were obtained from Life Technologies unless otherwise specified. 24-well glass-bottom cell culture plates were pre-coated with poly-D-lysine (0.2 mg/ml, Sigma) for 2 h at 37°C and washed with PBS. Hippocampal neurons were dissected from embryonic day 18 (E18) rats, dissociated with papain and DNaseI, and then plated at a density of 30,000 per cm^2^ in Neurobasal medium supplemented with B27, 2 mM GlutaMAX, 1% FBS. Cultures were maintained at 37°C in 5% carbon dioxide (CO_2_) and 100% humidity. Entire medium was replaced on 1DIV and refreshed 50% once on 8DIV. Neurons were transfected on 9DIV using Lipofectamine 2000 transfection kit (Clontech) following the manufacture’s instruction. 0.5 μg DNA was used for each well of neurons in a 24-well plate. All animal procedures were approved by the Institutional Animal Care and Use Committee at Stanford University and Chinese Academy of Sciences, Shanghai.

Characterization of Crimson in neurons. Live cell photobleaching was performed on neurons using an inverted wide-field microscope (Zeiss, Axiovert 200). Prior to the experiment, culture medium was replaced with imaging solution (HBSS + Hepes + L-glutamate). Cells were placed in a temperature chamber heated up to 37 C with 5% CO_2_, and continuously excited using Xenon Arc lamp filtered at 568/20 nm with a 20 ×0.75 NA objective lens, and images were detected with an emission filter at 620/60 nm. Time-series images were acquired every 10 s for each variant until the initial intensity was decreased by at least 40%.

Hippocampal neurons were transfected on 9 DIV and imaged on 12 DIV for brightness and aggregation measurements, and 18 DIV (cytosolic RFP-transfected neurons) or 15 DIV (membrane-bound RFP-transfected neurons) for viability measurements.

For brightness measurements, RFPs were imaged with a confocal scanning microscope (Leica, SP8), with a 585-nm laser whose power was set to a fixed level that captured all signals without saturation. At multiple positions, stacks of 40 sections with 0.5-μm spacing were acquired and then z-projected to create maximal intensity images. For cytosolic RFPs, 6 to 10 fields were imaged as technical replicates and a single mean intensity per cell area was calculated, and the experiment was repeated 3 times. For RFP-CAAX fusions, 5 to 8 fields were imaged as technical replicates and a single mean intensity per cell area was calculated, and the experiment was repeated 3 times. For viability measurements, a 10 × 10 grid of images was captured with a 10× lens to cover the inscribed square area of one well on a 24-well glass-bottom cell culture plate, and the experiment was repeated 3 times.

Image analysis. For brightness analysis, background was subtracted from each image. To quantify the brightness of RFPs in the whole cell, ImageJ was used to create a mask representing the contour of neurons with a threshold showing the majority of neurites, and the region of interest (ROI) was generated from the mask. The same criteria were applied to all images. The mean grey value within the ROI was then measured and divided by the ROI area to calculate brightness per cell area. For viability counting, neurons were assessed in a blinded manner.

Toxicity counting. The percentage of healthy neurons versus the total amount of transfected neurons was quantified to evaluate toxicity. Healthy neurons were defined as those with normal neuronal morphology lacking broken neurites or blebbing. Unhealthy neurons were defined as those with round cell bodies but broken neurites or blebbing. Dead neurons were defined as those with irregular or fragmented cell bodies and extensively fragmented neurites (Keskitalo et al., 2014; Zhang et al., 2014). Only transfected neurons were quantified to minimize the bias of transfection efficiency between different constructs.

Statistical analysis. Each biological replicate produced one data point per condition. The normality of data was confirmed using the Wilk-Shapiro test, then a single-factor ANOVA test was performed, with pairwise differences between Crimson and each other RFP assessed using the Dunnett post-hoc test.

Comparison of Crimson-CAAX and cytosolic mTurquoise2. For 1-photon imaging, cultured rat hippocampal neurons were transfected with a bi-directional vector co-expressing Crimson-CAAX and mTurquoise2 at 9DIV and imaged at 14–15DIV using either an epifluorescence microscope (Zeiss, Axiovert 200) with excitation at 440/10 nm and 568/20 nm and emission at 472/30 nm and 620/60 nm or using a confocal microscope (Leica, SP8) with excitation at 440 and 585 nm and emission at 450–510 nm and 595–675 nm. For 2-photon imaging, a Tol2 vector co-expressing mTurquoise2 and Crimson-CAAX was injected into the zebrafish embryos at 1-cell stage. Larval zebrafish with identifiable fluorescence at 7 days post-fertilization were paralyzed in 1 μm/μL α-bungarotoxin (Tocris) and mounted on their side in 2% low-melting-point agarose (Sigma). Two-photon fluorescence images (1024 × 1024 pixels) were obtained with an Olympus FVMPE multiphoton laser scanning microscope (Olympus), and a 25× objective (1.05 NA; Olympus) at 3×-18× optical zoom, 8–12.5 μs/pixel dwell times, and 3-5 frame averages. Fluorophores were excited at 1030 nm and emission was detected simultaneously with red and green channels.

Crystallization and structure solution of Crimson0.9. Prior to crystallization, hexahistidine-Crimson0.9 was purified using size-exclusion chromatography to remove aggregation. Thereafter, Crimson0.9 was buffer-exchanged with 50 mM Tris (hydroxymethyl) aminomethane, 25 mM NaCl, 4.0 mM TCEP at pH 7.4, and concentrated to 10 mg/ml for crystallization. Crimson0.9 was crystallized at 12°C in a dark chamber by sitting-drop vapor diffusion using 1 μL of 10 mg/ml Crimson0.9 in 50 mM Tris Base, 25 mM NaCl, 4.0 mM TCEP pH 7.4 mixed with 1 μL of 0.2 M MgCl_2_, 0.1 M Bis-Tris HCl pH 6.5, and 25% PEG 3350 and 1 μL of 1 M glycerol. Crimson0.9 crystals were cryoprotected in paratone oil and flash-frozen in liquid nitrogen. X-ray diffraction data were collected at the Stanford Synchrotron Radiation Laboratory on Beamline 12–2. The crystal structure was solved by molecular replacement using Phaser as part of the CCP4 suite55. Models of Crimson0.9 and the fluorophore were built with the Coot program. Refinement to 2.04 Å was performed in the REFMAC5 program with model rebuilding in Coot. Water molecules were manually added by inspection throughout the refinement process. The final model is composed of xx water molecules and all residues except residues 1–4 and 233–244 of chain A, 1–6 and 233–244 of chain B, 1–6 and 232–244 of chain C, 1–7 and 232–244 of chain D, as these did not show interpretable electron density. The quality of the model was then analyzed using the programs MolProbity and PROCHECK. The coordinates and reflections are deposited in the PDB with accession code 6MKP.

## Results

### Development of a Bright red Fluorescent Protein

**FIGURE 1:**
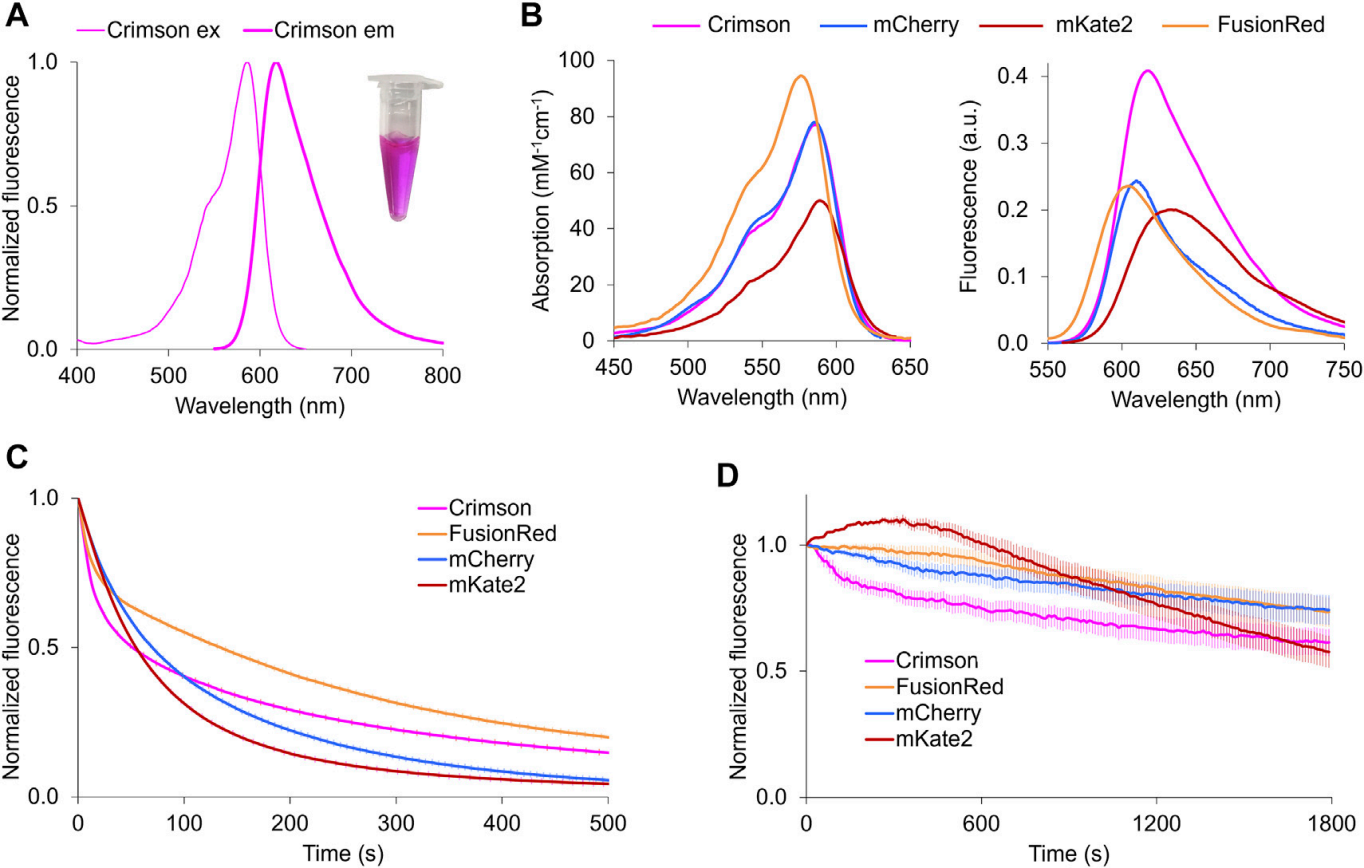
Characterization of Crimson *in vitro* and neurons. **(A)** Fluorescence spectra of Crimson. Inset, purified Crimson in visible light. **(B)** Absorption (left) and emission (right) of RFPs with excitation maxima at 580–590 nm. Absorbance spectra are scaled to peak extinction coefficient. Emission spectra are scaled so that areas under the curves are proportional to peak brightness (product of peak extinction coefficient and quantum yield). **(C)** Photobleaching kinetics of purified RFPs under arc lamp illumination with a 568/20-nm excitation filter. Time is scaled so that emission is normalized to 1000 photons per s. Imaging interval = 1 s. Each curve is the mean of two independent experiments with the error bars denoting SD (standard deviation, *n* ≥ 6). **(D)** Photobleaching of RFPs in transfected neurons under 585-nm laser illumination. Fluorescent intensity of each time frame is subtracted against the background and normalized to time point 0. Imaging interval = 10 s. Each curve is the mean with the error bars denoting SD (*n* = 3).

To obtain a bright RFP with similar excitation and emission spectra with mCherry, we performed structure-directed mutagenesis of mNeptune2, a bright and fast-maturing far-red fluorescent protein (Chu et al., 2014). We first performed mutagenesis on positions 11, 13, 28, and 41 to optimize the hydrogen bond interaction with the chromophore acylimine oxygen. After screening, a blue-shifted variant was obtained with best performance which carried three-point mutations at position 11, 28, and 41 (mNeptune2-M11S/S28H/G41N), while position 13 remained the same as the parent. We then introduced mutations into the outer barrel, the inner barrel and the loop respectively, in order to further blue-shift fluorescence spectra and optimize folding, maturation, photostability and brightness (Supplementary Figure S1 and Supplementary Table S1). After several rounds of screening, we obtained a bright RFP with peak excitation/emission at 588/617 nm (Figure 1A). This protein, named Crimson, differs from mNeptune2 by 33 mutations (13 inside the barrel) and 3 deletions (Supplementary Figure S1). Of these, 9 mutations mainly account for the spectral characteristics of Crimson (Supplementary Figure S2).

Crimson’s extinction coefficient (EC) of 77 mM^−1^cm^−1^ and quantum yield (QY) of 0.42 makes it ∼100% brighter than mCherry and FusionRed and 28% brighter than mKate2 per mature molecule (Table 1). Crimson’s absorption spectrum is similar to that of mCherry and mKate2, while its emission peak lies between those of mCherry and mKate2 (Figure 1B). Both absorption and emission spectra are redder for Crimson than for FusionRed (Figure 1B). Crimson exhibits reduced formation of a green fluorescent side-product common to eqFP578-derived RFPs (Supplementary Figure S3A), and is more pH-resistant than mKate2 (Supplementary Figure S3B, Table 1). Crimson exhibited bi-exponential fluorescence decay under one-photon widefield microscopy *in vitro*, with a faster initial drop to 50% brightness but slower subsequent photobleaching compared to mCherry, mKate2, and FusionRed (Figure 1C). Under laser illumination of cells expressing each RFP, Crimson again exhibited a fast initial drop, followed by photobleaching at a rate similar to mCherry and slower than mKate2 and FusionRed (Figure 1D). Similar to mKate2, Crimson is dimeric at 10 µM (Supplementary Figure S3C), consistent with minimal change to the dimeric interface during the evolution of these proteins from TagRFP. Crimson exhibited maturation speed and efficiency at 37°C comparable to mCherry and mKate2 and superior to FusionRed (Table 1).

**TABLE 1:** Key characteristics of red fluorescent proteins with excitation peak at 580–590 nm.

	Crimson	mCherry	mKate2	FusionRed
Ex peak (nm)	588	587	588	580
Em peak (nm)	617	610	633	608
EC (mM^−1^cm^−1^)	77	72	63	95
QY	0.42	0.22	0.4	0.19
Brightness^a^	32	16	25	18
Photostability (s)^b^	49	68	58	131
Maturation half-time (min)^c^	14	15	< 20	130
Maturation efficiency^d^	50%	44%	49%	24%
pKa	4.2	< 4.5	5.4	4.6
Oligomerization	dimer	monomer	dimer	monomer

a Calculated as the product of QY at peak excitation and EC in units of mM^−1^ cm^−1^.

b Predicted time for fluorescence to photobleach by 50% under arc-lamp illumination with excitation intensity adjusted to produce 1,000 emission photons per molecule per second.

c Time for fluorescence to obtain half-maximal value after exposure to oxygen.

d Functional chromophore concentration divided by total protein concentration. Functional chromophore is determined using the base-denaturation method as EC measurement. This excludes unfolded and broken-chromophore (backbone cleavage before the first residue of chromophore) components. Total protein is determined by absorbance at 280 nm.

Crimson’s high EC, high QY, and large emission separation from GFPs may make it a good Förster resonance energy transfer (FRET) acceptor for GFPs, especially when detecting FRET using sensitized emission (Bajar et al., 2016b). We thus assessed the performance of Crimson as a FRET acceptor compared to other RFPs including mRuby3 (Bajar et al., 2016a) and mScarlet-I (Bindels et al., 2017), two bright and blue-shifted monomeric RFPs. Indeed when fused to the bright GFP dClover2 (Bajar et al., 2016a), Crimson produced more efficient FRET than all tested RFPs and a higher RFP/GFP peak emission ratio than mCherry, mKate2, or FusionRed (Supplementary Figure S4). Incidentally, dClover2-mRuby3, dClover2-mScarlet-I, and dClover2-FusionRed would be predicted to exhibit more efficient FRET based on their longer Förster radii (r_0_) calculated from spectral characteristics of mature proteins, so the higher FRET efficiency of dClover2-Crimson also suggests superior maturation of Crimson. In mammalian cells, a Raichu RhoA reporter using dClover2-Crimson as the FRET pair demonstrated a larger emission ratio change upon activation than the one using dClover2-mRuby3 (Supplementary Figure S4). These results indicate that Crimson functions well as a FRET acceptor for GFPs. However, since Crimson is dimeric, it is not suitable for FRET applications involving fusion of Crimson to a cellular protein, such as protein-protein interaction and protein conformational changes, because this forced dimer may interfere with function or location of protein of interest.

### Structural Basis of Spectral Tuning in Crimson

**FIGURE 2:**
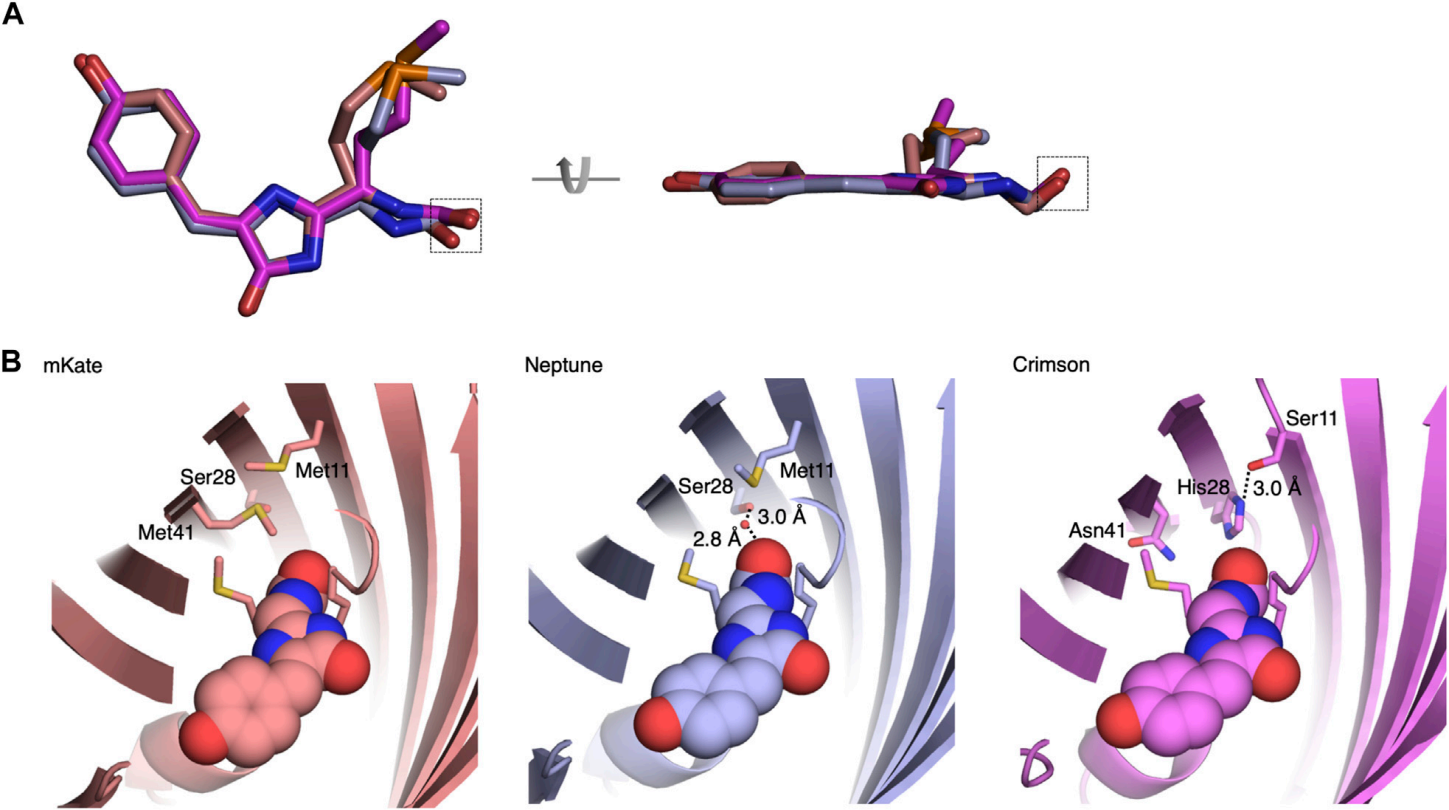
Structural basis of blue-shifting for Asn41, His28, and Ser11 in Crimson. **(A)** Alignment of Crimson (magenta), Neptune (PDB entry 3IP2, light blue) and mKate (PDB entry 3BXC, pink) chromophores. The chromophore rings are more coplanar in Crimson or Neptune than in mKate. The chromophore acylimine oxygen is indicated by the dashed box. **(B)** Hydrogen-bond interactions between the chromophore acylimine oxygen and its surrounding residues in Crimson, mKate and Neptune. In mKate, the acylimine oxygen does not engage in any hydrogen bond interactions. In Neptune, a water molecule donates a hydrogen bond to the acylimine oxygen and accepts a hydrogen bond from Ser-28. In Crimson, a hydrogen bond between Ser-11 and His-28 precludes one between His-28 and the acylimine oxygen. In addition, the side chain of Asn-41 is too short to hydrogen bond to the acylimine oxygen.

To understand the mechanism of spectral tuning in Crimson, we determined the atomic structure of a spectrally identical evolutionary predecessor, Crimson0.9, at a resolution of 2.0 Å at pH 6.7–6.8 (Supplementary Table S2). The crystallographic unit of Crimson0.9 contains four monomers with two parallel and two cross-dimerization interfaces. The positions of the chromophore and nearby amino acid side chains were similar in the four monomers (Supplementary Figure S5). As expected, each monomer has a typical eleven-stranded β-barrel structure with a central α-helix containing the covalently attached chromophore in the *cis* conformation **(** Figure 2, Supplementary Figure S5).

The Crimson0.9 structure reveals both similarities to and differences from the parental protein Neptune. Crimson0.9 retains the more planar chromophore conformation of Neptune relative to mKate, which may contribute to its higher extinction coefficient and quantum yield relative to mKate (Figure 2A). The primary structural change from the parental Neptune to Crimson0.9 is the loss of a hydrogen bond to the chromophore acylimine oxygen. In Neptune, this hydrogen bond is responsible for the excitation and emission red-shift relative to its predecessor mKate (Lin et al., 2009). Gly-41 of Neptune, whose lack of a side chain allows room for the water molecule, is mutated in Crimson0.9 to Asn-41. In mCardinal (Chu et al., 2014), a redder Neptune variant, Gln-41 serves as a hydrogen bond donor for the acylimine oxygen, but the side chain of Asn-41 of Crimson0.9 is too short to perform a similar function (Figure 2B). In addition, Ser-28, which assists in holding the water molecule in place in Neptune, is replaced with the larger His-28 in Crimson0.9, occluding water from the chromophore acylimine vicinity **(** Figure 2B). The electron density of His-28 in the Crimson0.9 crystal is consistent with two rotamers with different and mutually exclusive hydrogen-bonding patterns: in one, the Nε atom donates a hydrogen bond to the chromophore and the Nδ atom lacks hydrogen bond partners, while in the other, the Nε is hydrogen-bonded to Ser-11 and Nδ lacks hydrogen bond partners (Figure 2B). However, the electron density of Ser-11 indicates that its hydroxyl group is oriented toward His-28 despite not being hindered from adopting alternative conformations; this suggests that His-28 indeed is engaged in a hydrogen bond with only Ser-11.

Finally, His-28 is unlikely to exert any electrostatic influence on chromophore electronic distributions, as it is predicted to be uncharged at physiological pH by the PROPKA3.0 program. Thus, the acylimine group of Crimson both lacks hydrogen bonding and exists within a neutral environment. These features are shared between Crimson and mKate (Figure 2), and would be consistent with the similarity of their spectra (Figure 1). Thus, the M11S, S28H, and G41N mutations acquired in the evolution of Crimson from Neptune essentially undo the red-shifting effect of the M41G mutation acquired in the evolution of Neptune from mKate, while allowing for higher molecular brightness.

### Comparison of Cytosolic Red Fluorescent Proteins for Brightness, Aggregation, and Toxicity in Neurons

**FIGURE 3:**
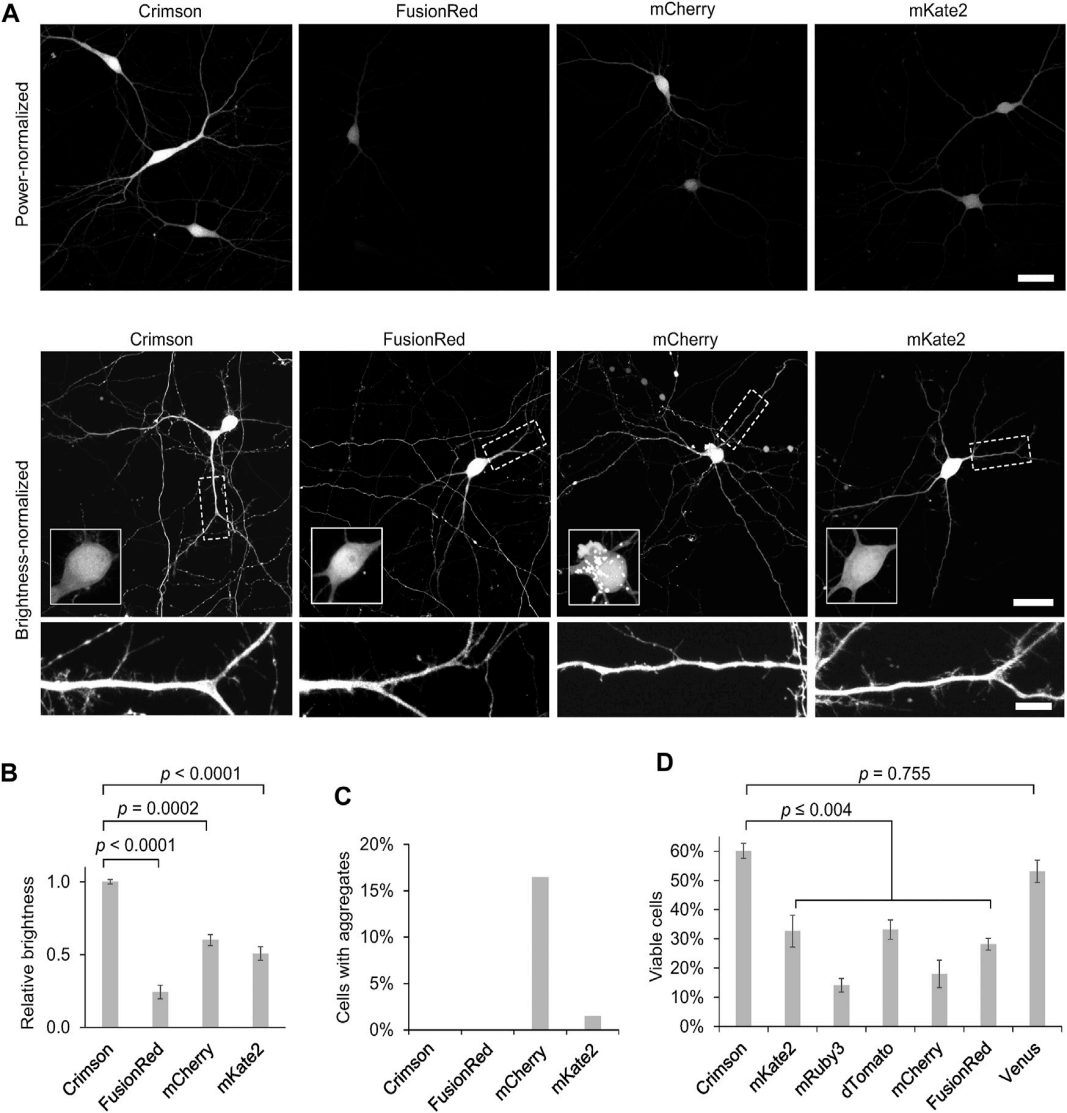
Cytosolic Crimson is brighter and less cytotoxic than other RFPs in neurons. **(A)** Comparison of RFPs in rat hippocampal neurons imaged with confocal microscopy 3 days post-transfection at 12 DIV. Top, representative images of transfected neurons acquired and displayed with identical settings are shown. Scale bar = 20 µm. Bottom, representative images with brightness adjusted to similar levels for display. Cell bodies are enlarged in the insets within the upper panels. Dendritic segments indicated by the dashed rectangle are enlarged in the lower panels. Scale bar = 5 µm. **(B)** Quantification of RFP brightness 3 days post-transfection at 12 DIV. Error bars = SEM (Standard Error of the Mean). Overall *p* < 0.0001 by one-factor ANOVA. **(C)** Quantification of the percentage of neurons with aggregates among total transfected neurons 3 days post-transfection at 12 DIV. **(D)** Quantification of the percentage of healthy neurons among total transfected neurons 9 days post-transfection at 18 DIV. Error bars = SEM. Overall *p* < 0.0001 by one-factor ANOVA.

As improved RFPs are especially needed in neuronal applications, we compared the performance of Crimson and other RFPs in cultured primary hippocampal neurons. While measurements of extinction coefficient and quantum yield on purified proteins *in vitro* allow an objective measurement of per-molecule brightness of mature fluorescent protein, apparent brightness of fluorescent protein constructs in cells is also influenced by the efficiency of protein maturation and folding, the half-life of the protein and even cell types may cause variations; thus such characteristics should be empirically tested. We thus expressed Crimson, FusionRed, mCherry, and mKate2 in 9 days *in vitro* (DIV) cultured rat hippocampal neurons. After 3 days of expression (12 DIV), Crimson was significantly brighter than the other RFPs when imaged under the same conditions (Figure 3A), with a whole-cell-brightness 4.3-, 1.6-, and 2-fold of that of FusionRed, mCherry, and mKate2, respectively (Figure 3B).

We noticed large aggregates in the cell bodies of mCherry-transfected neurons (Figure 3A, Figure 3C). As protein aggregates could lead to neuronal death after long-term expression, we next measured the ability of neurons to survive over time when expressing various fluorescent proteins. In addition to the above RFPs, we also tested the effects of chronic expression of mRuby3, dTomato, and the YFP Venus. We added mRuby3 to test a monomeric RFP with higher brightness than mCherry, dTomato to determine if expression of a strongly dimeric RFP might actually be the least toxic by reducing the concentration of free monomers with an unbound dimerization interface, and Venus as a widely used derivative of *Aequoria victoria* GFP. After 9 days of expression (at 18 DIV), Crimson-expressing neurons demonstrated significantly higher survival rates than neurons expressing the other RFPs, and similar survival rates as neurons expressing low-toxicity Venus (Figure 3D). Interestingly, dTomato was as toxic as mKate2 and more toxic than Crimson, suggesting that strong dimer affinity is not the main mechanism for the reduced toxicity of Crimson. On the other hand, the strong monomers mCherry and FusionRed also were more toxic than Crimson, suggesting that monomerization alone is also not correlated with survival. Regardless, Crimson performs better than the spectrally similar mCherry, mKate2, and FusionRed fully visualizing the dendritic processes of neurons with improved brightness and reduced toxicity.

### Comparison of Membrane-Tethered Red Fluorescent Proteins in Neurons

**FIGURE 4:**
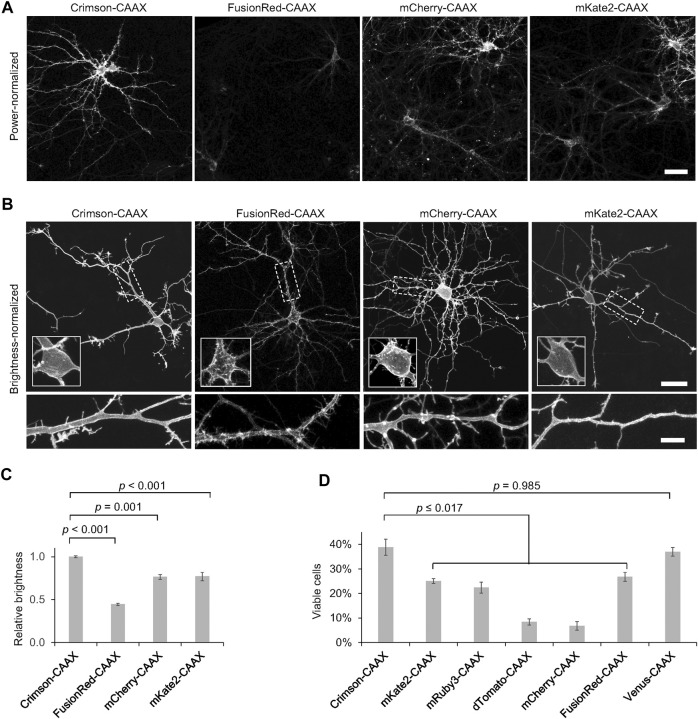
Crimson-CAAX labels the cytoplasmic membrane well in neurons. **(A)** Brightness comparison of RFP-CAAX fusions in rat hippocampal neurons 3 days post-transfection at 12 DIV. Representative confocal images of transfected neurons acquired and displayed with identical settings are shown. Scale bar = 20 µm. **(B)** RFP-CAAX fusions visualized with confocal microscopy 3 days post-transfection at 12 DIV, with image brightness adjusted to similar levels for display. Cell bodies are enlarged in the insets within the upper panels. Dendritic segments indicated by the dashed rectangle are enlarged in the lower panels. Scale bar = 20 µm (upper panels) or 5 µm (lower panels). **(C)** Quantification of brightness of RFP-CAAX constructs 3 days post-transfection at 12 DIV. Error bars = SEM. Overall *p* < 0.001 by one-factor ANOVA. **(D)** Quantification of the percentage of healthy neurons among total transfected neurons 6 days post-transfection at 15 DIV. Crimson-CAAX-expressing neurons demonstrated the highest viability. Error bars = SEM. Overall *p* < 0.0001 by one-factor ANOVA.

To determine the utility of RFPs for labeling the neuronal plasma membrane, we transfected neurons with a series of RFP fusions to the CAAX farnesylation motif and quantified the brightness of each RFP-CAAX reporter. We observed that Crimson-CAAX was significantly brighter than mKate2-CAAX, mCherry-CAAX, or FusionRed-CAAX (Figures 4A,C). We then adjusted excitation power to equalize brightness across RFPs to visualize fluorescence distributions within neurons. While mKate2-CAAX, FusionRed-CAAX and Crimson-CAAX distributed along the plasma membrane and evenly labelled neurites and dendritic spines, mCherry-CAAX accumulated in aggregates within dendritic shafts (Figure 4B).

We then assessed the viability of neurons expressing the same set of RFPs previously tested for toxicity, but now fused to the CAAX motif. Crimson-CAAX exhibited significantly higher survival rates than neurons expressing the other RFP-CAAX fusions, and similar survival rates as neurons expressing a Venus-CAAX fusion (Figure 4D). Neurons expressing dTomato-CAAX and mCherry-CAAX showed the least viability. Strikingly, dTomato-CAAX created dramatic rod-like structures in neurons (Supplementary Figure S6). This suggests that dTomato may contain an interface capable of polymerization once dTomato is concentrated and oriented at the membrane by farnesylation.

### Crimson-CAAX in Imaging Dendritic Spines *Ex Vivo* and *in vivo*

**FIGURE 5:**
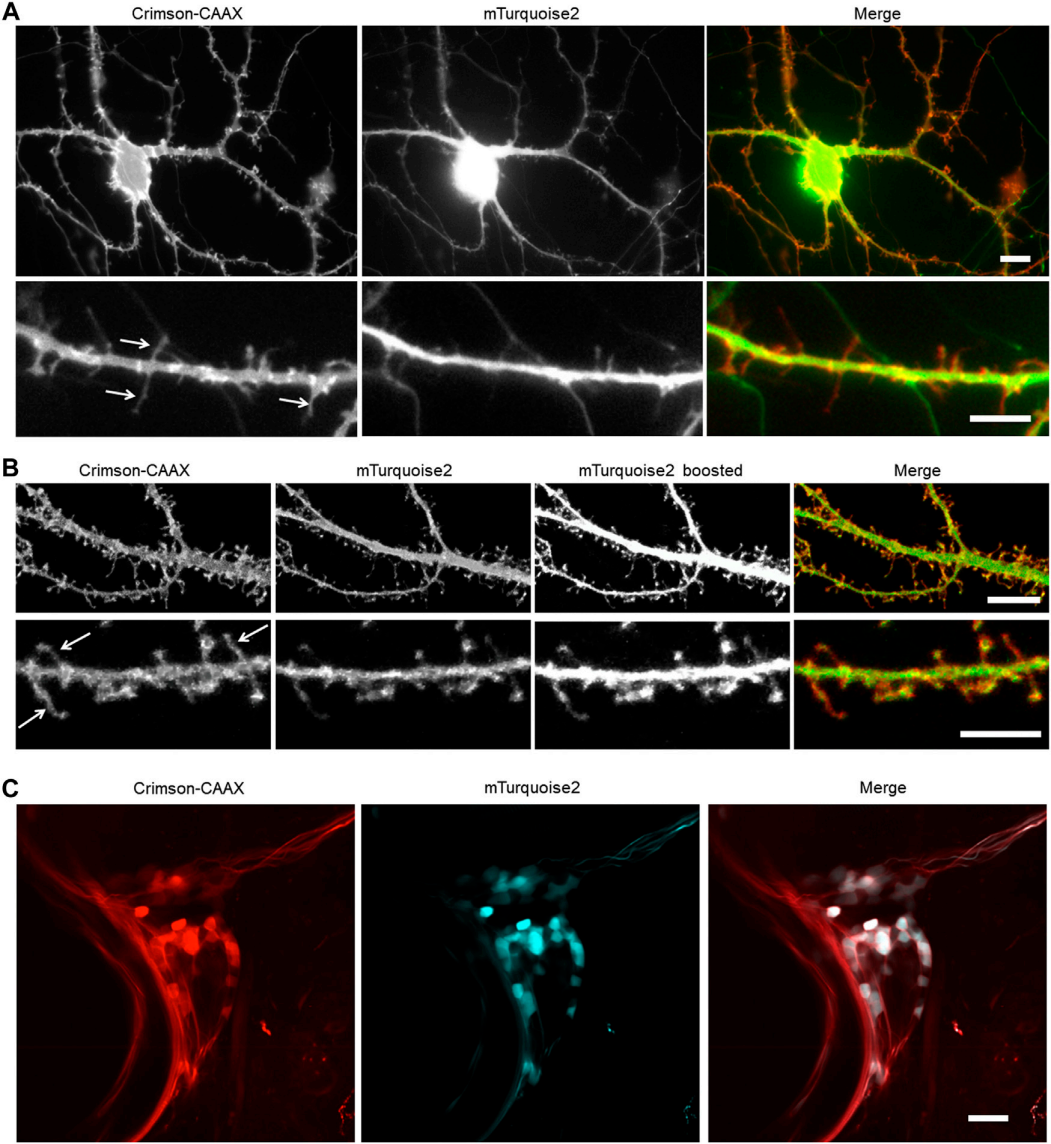
Membrane-bound Crimson improves the detection of small processes. **(A,B)** Visualization of cultured rat hippocampal neurons with an epifluorescent microscope **(A)** and a confocal microscope **(B)**. Neurons were co-transfected with cytosolic mTurquoise2 and Crimson-CAAX at 9 DIV and imaged at 14 DIV. Lower panels show dendrites from the same culture at higher magnification. Arrows indicate thin spines and filopodia visible with Crimson-CAAX but not with cytosolic mTurquoise2. Scale bars = 10 µm (upper panels) and 5 µm (lower panels). **(C)** Visualization of zebrafish trigeminal ganglion by two-photon microscopy *in vivo*. Zebrafish was injected with DNA at one-cell stage and imaged at 7-day post-fertilization. Scale bar = 20 µm.

We hypothesized that Crimson-CAAX would allow more sensitive detection of small membrane structures such as filopodia and spines than a cytosolic fluorescent protein, due to the low amount of cytosol in these small structures. To compare Crimson-CAAX with a cytosolic fill FP, we co-expressed Crimson-CAAX with cytosolic mTurquoise2 in primary hippocampal neurons. In standard wide-field epifluorescence microscopy, Crimson-CAAX distributed evenly in soma and the neuronal processes, and clearly labeled thin dendritic processes (Figure 5A). In contrast, cytosolic mTurquoise2 was mostly detected in the soma and the proximal portions of the dendrites and did not label the thinner dendritic processes as clearly (Figure 5A, upper panels). At high magnification, Crimson-CAAX could be seen along the membrane of spines and filopodia, with similar brightness per membrane length compared to nearby dendritic membranes. In contrast, cytosolic mTurqouise2 produced signals that were orders of magnitude dimmer in spines than in adjacent dendritic shafts, causing scatter from dendritic shafts to reduce the signal-to-background ratio for detection of spines and filopodia (Figure 5A, lower panels).

Crimson-CAAX also performed better than mTurquoise2 in labeling thin structures in optical-sectioning microscopy. In confocal microscopy, filopodia and spines became visible in the cytosolic mTurquoise2 channel only when the brightness was boosted and part of the dendrite shafts became overexposed, whereas filopodia, spines, and dendritic shafts could be visualized with similar intensities in the Crimson-CAAX channel (Figure 5B). We also tested Crimson-CAAX versus cytosolic mTurquoise2 in the neurons of living zebrafish under two-photon illumination. Zebrafish dendrites are small in diameter, measuring <3 μm at their widest point, compared to 5–10 μm of mammalian neurons. In the trigeminal ganglion and developing spinal cord, cytosolic mTurquoise2 was only visible in a small fraction of Crimson-positive neurites, even when using laser and detector settings that produced more mTurquoise2 than Crimson-CAAX signal intensity in cell bodies (Figure 5C). Thus, in one-photon widefield, one-photon confocal, and two-photon microscopy, Crimson-CAAX improved the detection of filopodia and spines.

## Discussion

In this study, we developed a new bright RFP, Crimson, with similar excitation and emission spectra as the widely used mCherry and mKate2. In neurons, Crimson is brighter and less toxic than mKate2, mCherry, or FusionRed, either when expressed in the cytosol or targeted to the membrane. A membrane-targeted form of Crimson improves the visualization of spines and filopodia, which contain minuscule amounts of cytoplasm, compared to a cytosolic fluorescent protein. In one-photon microscopy, Crimson-CAAX reliably visualizes filopodia and thin spine necks, which are only clearly visible with cytosolic FPs when the dendritic shafts are overexposed. In two-photon microscopy, Crimson-CAAX outperforms the cytosolic FP in labeling thin neurites. Thus, Crimson-CAAX may be useful for studying synaptic morphology in development (Yoshihara et al., 2009) or for sparse labeling and circuit tracing *in vivo* (Xu and Sudhof, 2013). It may be especially useful for testing interventions in models of neurodevelopmental disorders characterized by abnormal filopodia or spines (De Rubeis et al., 2013; Michaelsen-Preusse et al., 2016).

The fact that the weakly dimeric Crimson is less toxic to neurons than the more monomeric mCherry or FusionRed in both cytosolic and membrane-bound forms is surprising. This observation suggests that the presence of a dimeric interface is not necessarily deleterious to the cell. Crimson is also less toxic than the similarly dimeric mKate2 and the more dimeric dTomato, which suggests that dimerization is not necessarily protective either. Indeed, dTomato when targeted to the membrane creates dramatic rod-like structures in neurons and is highly toxic. Thus factors other than homodimerization affinity alone influence toxicity. One explanation could be that mutation of Crimson at 23 exterior-facing locations (Supplementary Figure S1) alters its interactions with endogenous neuronal proteins in a manner that reduces toxicity.

Certain tetrameric RFPs have previously been shown to be suitable for whole cell labeling (Strack et al., 2008; Strack et al., 2009). For example, DsRed-Express2 is reportedly bright, nontoxic and reasonably photostable (Strack et al., 2008). While it is possible for DsRed-Express2 to work with a CAAX signal to the extent that it can be targeted to the membrane, it may form aggregates at the cell membrane, as was observed in DsRed.T3-CAAX, the precursor of DsRed-Express2 (Schwirz et al., 2020). We have also observed that tdTomato-CAAX forms large rod-like structures (Supplementary Figure S6), presumably due to interaction between tdTomato-CAAX chains at the parallel dimerization interface (the two Tomato domains in tdTomato form a cross-dimer, leaving the parallel dimerization interface unoccupied). Therefore, tetrameric RFP-CAAX fusions would not be a preferred choice for membrane labelling.

In summary, Crimson is a bright RFPs that is uniquely useful for long-term imaging in neurons, either as a cytosolic protein or as a membrane-targeted fusion. It is brighter than mCherry, mKate2, and FusionRed, commonly used RFPs or far-red FPs in its spectral class, and it is also uniquely non-toxic to neurons after long-term expression. With these favorable characteristics, Crimson may be a favorable starting point for further engineering to create a fully monomeric version that is compatible with fusion to a variety of neuronal proteins.

## Data Availability

The original contributions presented in the study are included in the article/Supplementary Material, further inquiries can be directed to the corresponding authors.

## References

[B1] Bajar B. T. Wang E. S. Zhang S. Lin M. Z. Chu J. (2016a). A Guide to Fluorescent Protein FRET Pairs. Sensors (Basel) 16. 10.3390/s16091488 PMC5038762 27649177

[B2] Bajar B. T. Wang E. S. Lam A. J. Kim B. B. Jacobs C. L. Howe E. S. (2016b). Improving Brightness and Photostability of Green and Red Fluorescent Proteins for Live Cell Imaging and FRET Reporting. Sci. Rep. 6, 20889. 10.1038/srep20889 26879144 PMC4754705

[B3] Bindels D. S. Haarbosch L. Van Weeren L. Postma M. Wiese K. E. Mastop M. (2017). mScarlet: a Bright Monomeric Red Fluorescent Protein for Cellular Imaging. Nat. Methods 14, 53–56. 10.1038/nmeth.4074 27869816

[B4] Chu J. Haynes R. D. Corbel S. Y. Li P. González-González E. Burg J. S. (2014). Non-invasive Intravital Imaging of Cellular Differentiation with a Bright Red-Excitable Fluorescent Protein. Nat. Methods 11, 572–578. 10.1038/nmeth.2888 24633408 PMC4008650

[B5] Cranfill P. J. Sell B. R. Baird M. A. Allen J. R. Lavagnino Z. De Gruiter H. M. (2016). Quantitative Assessment of Fluorescent Proteins. Nat. Methods 13, 557–562. 10.1038/nmeth.3891 27240257 PMC4927352

[B6] Craske M. L. Fivaz M. Batada N. N. Meyer T. (2005). Spines and Neurite Branches Function as Geometric Attractors that Enhance Protein Kinase C Action. J. Cell Biol. 170, 1147–1158. 10.1083/jcb.200503118 16186260 PMC2171530

[B7] Day R. N. Davidson M. W. (2009). The Fluorescent Protein Palette: Tools for Cellular Imaging. Chem. Soc. Rev. 38, 2887–2921. 10.1039/b901966a 19771335 PMC2910338

[B8] De Rubeis S. Pasciuto E. Li K. W. Fernández E. Di Marino D. Buzzi A. (2013). CYFIP1 Coordinates mRNA Translation and Cytoskeleton Remodeling to Ensure Proper Dendritic Spine Formation. Neuron 79, 1169–1182. 10.1016/j.neuron.2013.06.039 24050404 PMC3781321

[B9] Feng J. Zhang C. Lischinsky J. E. Jing M. Zhou J. Wang H. (2019). A Genetically Encoded Fluorescent Sensor for Rapid and Specific *In Vivo* Detection of Norepinephrine. Neuron 102, 745–761. 10.1016/j.neuron.2019.02.037 30922875 PMC6533151

[B10] Fenno L. E. Ramakrishnan C. Kim Y. S. Evans K. E. Lo M. Vesuna S. (2020). Comprehensive Dual- and Triple-Feature Intersectional Single-Vector Delivery of Diverse Functional Payloads to Cells of Behaving Mammals. Neuron 107, 836–853. 10.1016/j.neuron.2020.06.003 32574559 PMC7687746

[B11] Govek E.-E. Newey S. E. Akerman C. J. Cross J. R. Van Der Veken L. Van Aelst L. (2004). The X-Linked Mental Retardation Protein Oligophrenin-1 Is Required for Dendritic Spine Morphogenesis. Nat. Neurosci. 7, 364–372. 10.1038/nn1210 15034583

[B12] Han C. Jan L. Y. Jan Y.-N. (2011). Enhancer-driven Membrane Markers for Analysis of Nonautonomous Mechanisms Reveal Neuron-Glia Interactions in Drosophila. Proc. Natl. Acad. Sci. U.S.A. 108, 9673–9678. 10.1073/pnas.1106386108 21606367 PMC3111288

[B13] Holtmaat A. Bonhoeffer T. Chow D. K. Chuckowree J. De Paola V. Hofer S. B. (2009). Long-term, High-Resolution Imaging in the Mouse Neocortex through a Chronic Cranial Window. Nat. Protoc. 4, 1128–1144. 10.1038/nprot.2009.89 19617885 PMC3072839

[B14] Keskitalo S. Farkas M. Hanenberg M. Szodorai A. Kulic L. Semmler A. (2014). Reciprocal Modulation of AÎ²42 Aggregation by Copper and Homocysteine. Front. Aging Neurosci. 6, 237. 10.3389/fnagi.2014.00237 25249976 PMC4157544

[B15] Laviv T. Kim B. B. Chu J. Lam A. J. Lin M. Z. Yasuda R. (2016). Simultaneous Dual-Color Fluorescence Lifetime Imaging with Novel Red-Shifted Fluorescent Proteins. Nat. Methods 13, 989–992. 10.1038/nmeth.4046 27798609 PMC5322478

[B16] Lin M. Z. Mckeown M. R. Ng H.-L. Aguilera T. A. Shaner N. C. Campbell R. E. (2009). Autofluorescent Proteins with Excitation in the Optical Window for Intravital Imaging in Mammals. Chem. Biol. 16, 1169–1179. 10.1016/j.chembiol.2009.10.009 19942140 PMC2814181

[B17] Michaelsen-Preusse K. Zessin S. Grigoryan G. Scharkowski F. Feuge J. Remus A. (2016). Neuronal Profilins in Health and Disease: Relevance for Spine Plasticity and Fragile X Syndrome. Proc. Natl. Acad. Sci. U.S.A. 113, 3365–3370. 10.1073/pnas.1516697113 26951674 PMC4812738

[B18] Ng H.-L. Lin M. Z. (2016). Structure-guided Wavelength Tuning in Far-Red Fluorescent Proteins. Curr. Opin. Struct. Biol. 39, 124–133. 10.1016/j.sbi.2016.07.010 27468111 PMC5548387

[B19] Pletnev S. Shcherbo D. Chudakov D. M. Pletneva N. Merzlyak E. M. Wlodawer A. (2008). A Crystallographic Study of Bright Far-Red Fluorescent Protein mKate Reveals pH-Induced Cis-Trans Isomerization of the Chromophore. J. Biol. Chem. 283, 28980–28987. 10.1074/jbc.m800599200 18682399 PMC2570900

[B20] Sala C. Segal M. (2014). Dendritic Spines: the Locus of Structural and Functional Plasticity. Physiol. Rev. 94, 141–188. 10.1152/physrev.00012.2013 24382885

[B21] Schwirz J. Yan Y. Franta Z. Schetelig M. F. (2020). Bicistronic Expression and Differential Localization of Proteins in Insect Cells and Drosophila Suzukii Using Picornaviral 2A Peptides. Insect Biochem. Mol. Biol. 119, 103324. 10.1016/j.ibmb.2020.103324 31978587

[B22] Shaner N. C. Campbell R. E. Steinbach P. A. Giepmans B. N. G. Palmer A. E. Tsien R. Y. (2004). Improved Monomeric Red, Orange and Yellow Fluorescent Proteins Derived from Discosoma Sp. Red Fluorescent Protein. Nat. Biotechnol. 22, 1567–1572. 10.1038/nbt1037 15558047

[B23] Shemiakina II Ermakova G. V. Cranfill P. J. Baird M. A. Evans R. A. Souslova E. A. (2012). A Monomeric Red Fluorescent Protein with Low Cytotoxicity. Nat. Commun. 3, 1204. 10.1038/ncomms2208 23149748

[B24] Shen Y. Lai T. Campbell R. E. (2015). Red Fluorescent Proteins (RFPs) and RFP-Based Biosensors for Neuronal Imaging Applications. Neurophoton 2, 031203. 10.1117/1.nph.2.3.031203 PMC4478792 26158012

[B25] St-Pierre F. Marshall J. D. Yang Y. Gong Y. Schnitzer M. J. Lin M. Z. (2014). High-fidelity Optical Reporting of Neuronal Electrical Activity with an Ultrafast Fluorescent Voltage Sensor. Nat. Neurosci. 17, 884–889. 10.1038/nn.3709 24755780 PMC4494739

[B26] Strack R. L. Hein B. Bhattacharyya D. Hell S. W. Keenan R. J. Glick B. S. (2009). A Rapidly Maturing Far-Red Derivative of DsRed-Express2 for Whole-Cell Labeling. Biochemistry 48, 8279–8281. 10.1021/bi900870u 19658435 PMC2861903

[B27] Strack R. L. Strongin D. E. Bhattacharyya D. Tao W. Berman A. Broxmeyer H. E. (2008). A Noncytotoxic DsRed Variant for Whole-Cell Labeling. Nat. Methods 5, 955–957. 10.1038/nmeth.1264 18953349 PMC4107390

[B28] Woolfrey K. M. Srivastava D. P. Photowala H. Yamashita M. Barbolina M. V. Cahill M. E. (2009). Epac2 Induces Synapse Remodeling and Depression and its Disease-Associated Forms Alter Spines. Nat. Neurosci. 12, 1275–1284. 10.1038/nn.2386 19734897 PMC2754861

[B29] Xu T. Yu X. Perlik A. J. Tobin W. F. Zweig J. A. Tennant K. (2009). Rapid Formation and Selective Stabilization of Synapses for Enduring Motor Memories. Nature 462, 915–919. 10.1038/nature08389 19946267 PMC2844762

[B30] Xu W. Südhof T. C. (2013). A Neural Circuit for Memory Specificity and Generalization. Science 339, 1290–1295. 10.1126/science.1229534 23493706 PMC3651700

[B31] Yoshihara Y. De Roo M. Muller D. (2009). Dendritic Spine Formation and Stabilization. Curr. Opin. Neurobiol. 19, 146–153. 10.1016/j.conb.2009.05.013 19523814

[B32] Zhang J. Sun X. Zheng S. Liu X. Jin J. Ren Y. (2014). Myelin Basic Protein Induces Neuron-specific Toxicity by Directly Damaging the Neuronal Plasma Membrane. PLoS One 9, e108646. 10.1371/journal.pone.0108646 25255088 PMC4177931

[B33] Zhou L. Jones E. V. Murai K. K. (2012). EphA Signaling Promotes Actin-Based Dendritic Spine Remodeling through Slingshot Phosphatase. J. Biol. Chem. 287, 9346–9359. 10.1074/jbc.m111.302802 22282498 PMC3308827

