# Supplementary material for: A Bright, Nontoxic, and Non-aggregating red Fluorescent Protein for Long-Term Labeling of Fine Structures in Neurons
Source: Front Cell Dev Biol. 2022 Jun 29;10:893468. doi: 10.3389/fcell.2022.893468 (PMC9278655; doi:10.3389/fcell.2022.893468)
Supplement: Supplementary file 1 [file DataSheet1.DOCX]

Supplementary Material

**
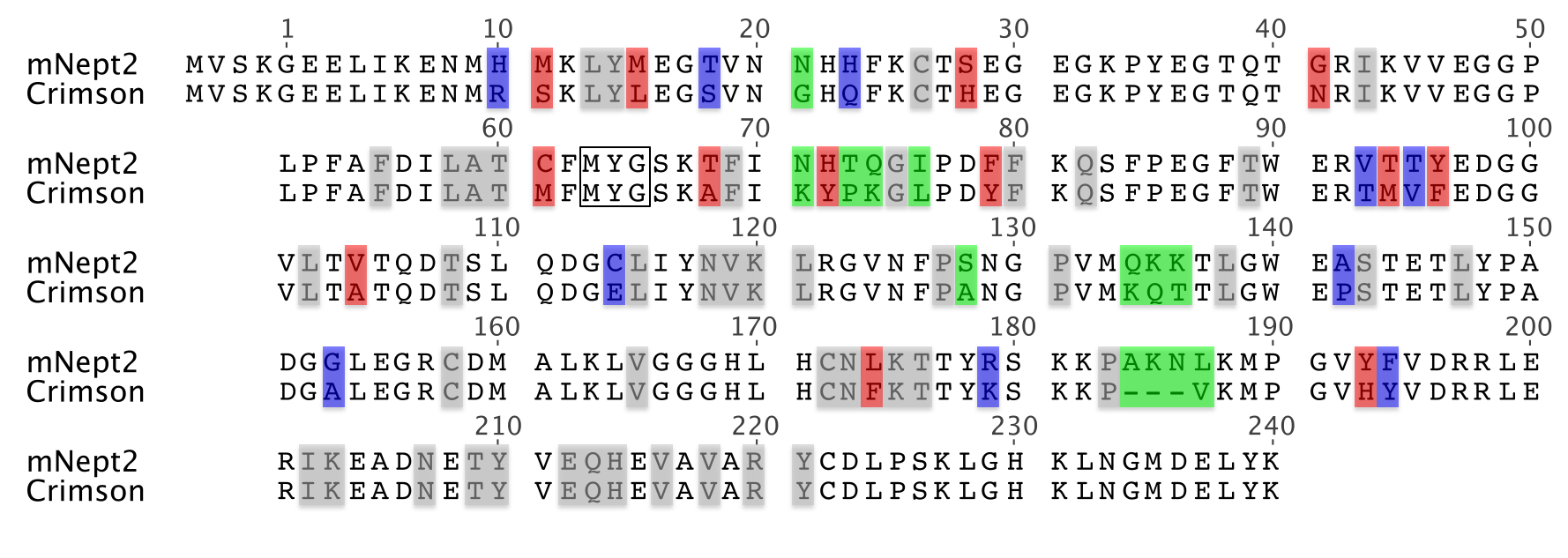
**

**Supplementary Figure 1. Protein sequence alignment of Crimson to mNeptune2.** The chromophore-forming residues are enclosed in a black box. Beneficial outer barrel mutations, inner barrel mutations and loop mutations are marked in blue, red and green, respectively. Residues shaded gray were mutated but not changed during evolution. All mutations and the rationale behind Crimson are summarized in Supplementary Table 1. The numbering is according to the crystal structure of mNeptune1 (PDB entry: 3IP2).


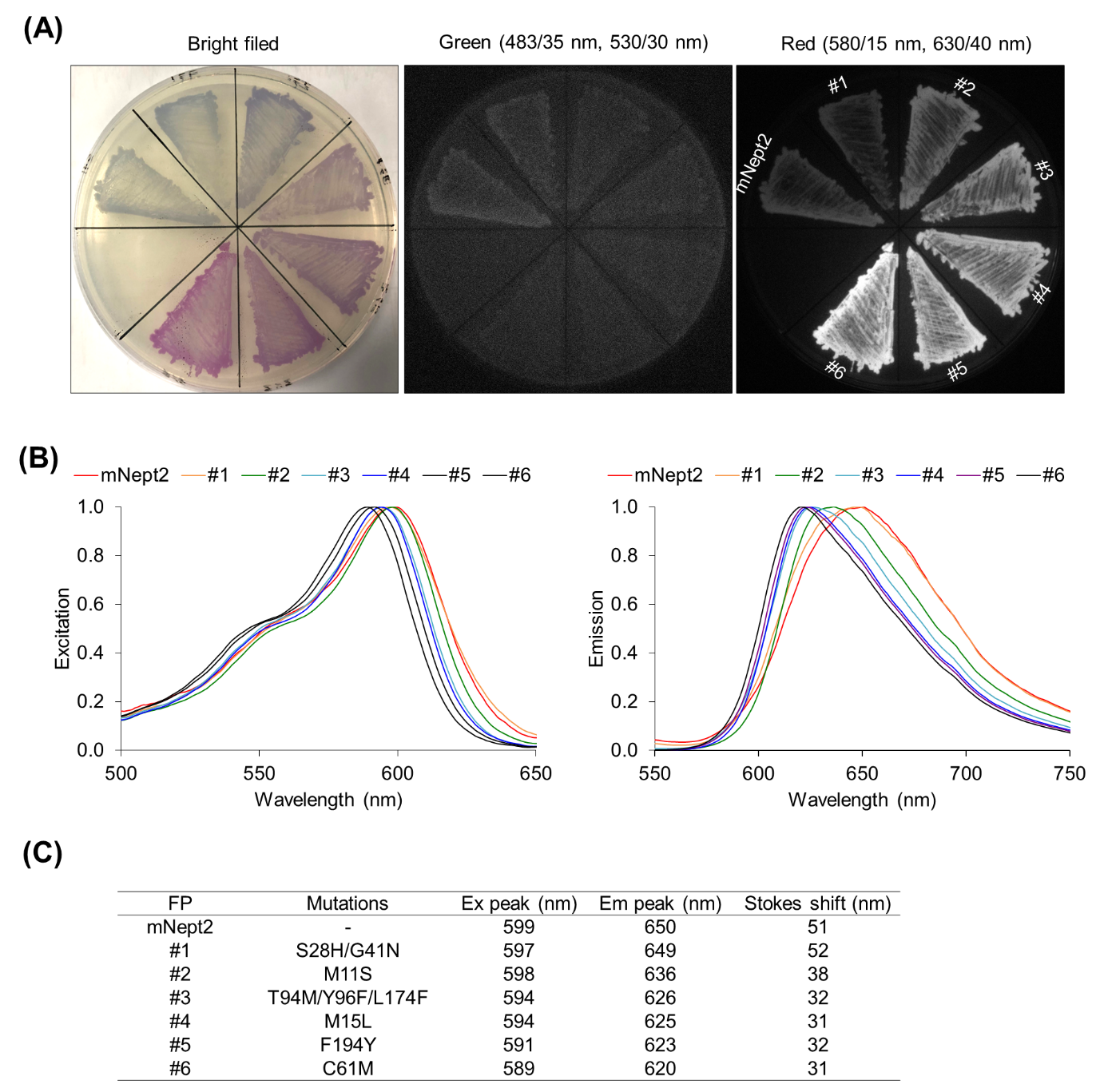


**Supplementary Figure 2. Residues contributing blue-shifting and small Stokes shift in Crimson. (A)** Bright-field (left) and fluorescence (middle and right) images of patches expressing mNeptune2 blue-shifted mutants. All patches were incubated at 34 °C for 20 h and kept at 4 °C for 48 h to ensure all RFPs fully matured. Fluorescence images were acquired with a home-made fluorescence system. **(B)** Excitation and emission spectra of mNeptune2 mutants. **(C)** Summary of mNeptune2 mutants presented in (B).


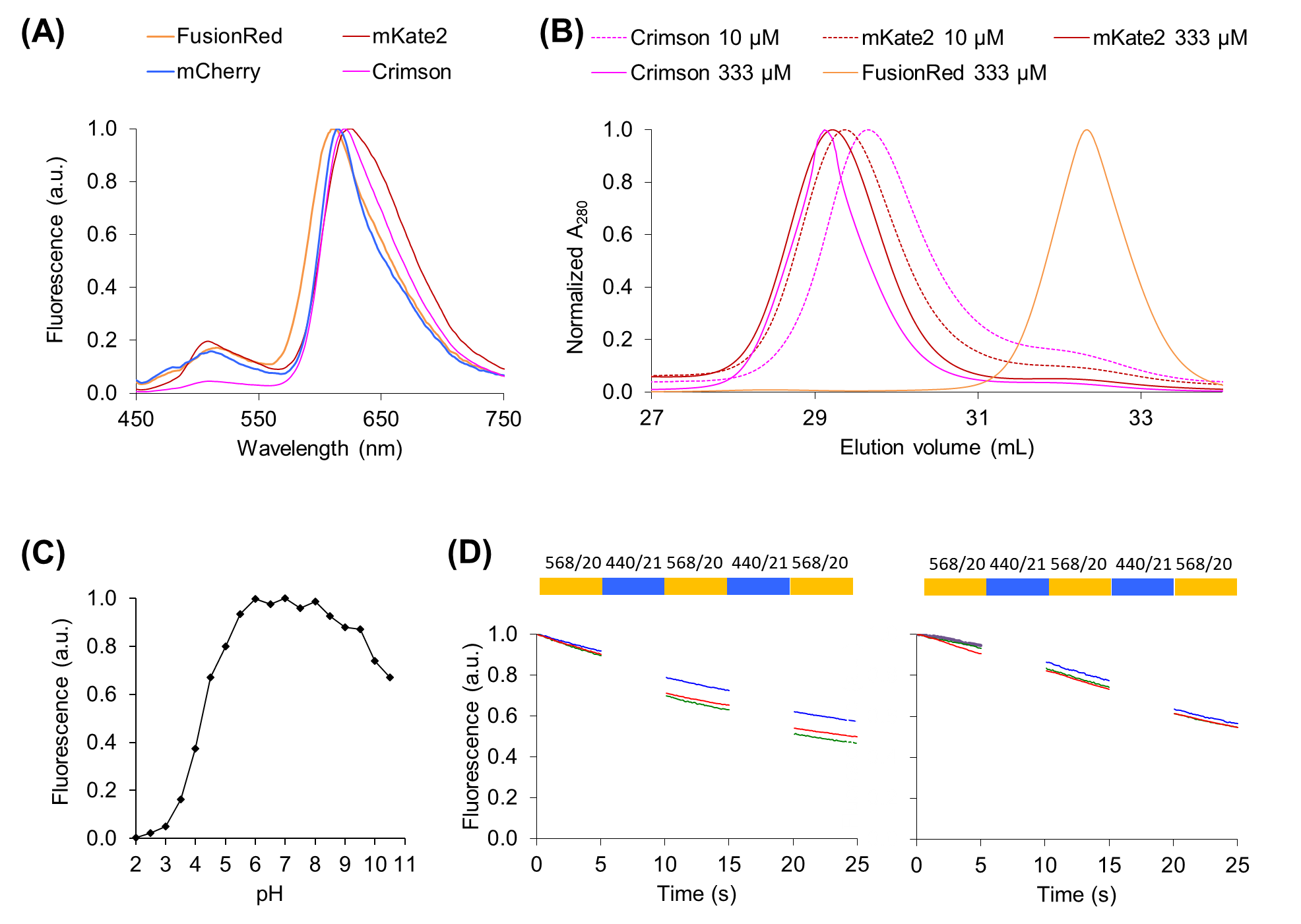


**Supplementary Figure 3. Additional characteristics of Crimson.** **(A)** Green components of RFPs in bacteria. Emission spectra were taken for bacteria (overnight growth at 36°C, 8h incubation at room temperature and 48h at 4°C) expressing RFPs when excited at 440 nm. **(B)** Gel filtration of Crimson at loading concentrations of 10 μM and 333 μM (10 mg/mL) reveals it to be dimeric even at 10 μM. **(C)** pH dependence of Crimson fluorescence showing a pKa of 4.2. **(D)** Photochromic behavior of mScarlet-I (left) and Crimson (right) in *E.coli*. Bacterial cells expressing RFPs were illuminated with alternating 5-sec exposures to 568/20 nm (21.85 W/cm^2^) and 440/21 nm (16.3 mW/cm^2^) light. Note: all powers were measured close to the back-focal plane of the objective lens.


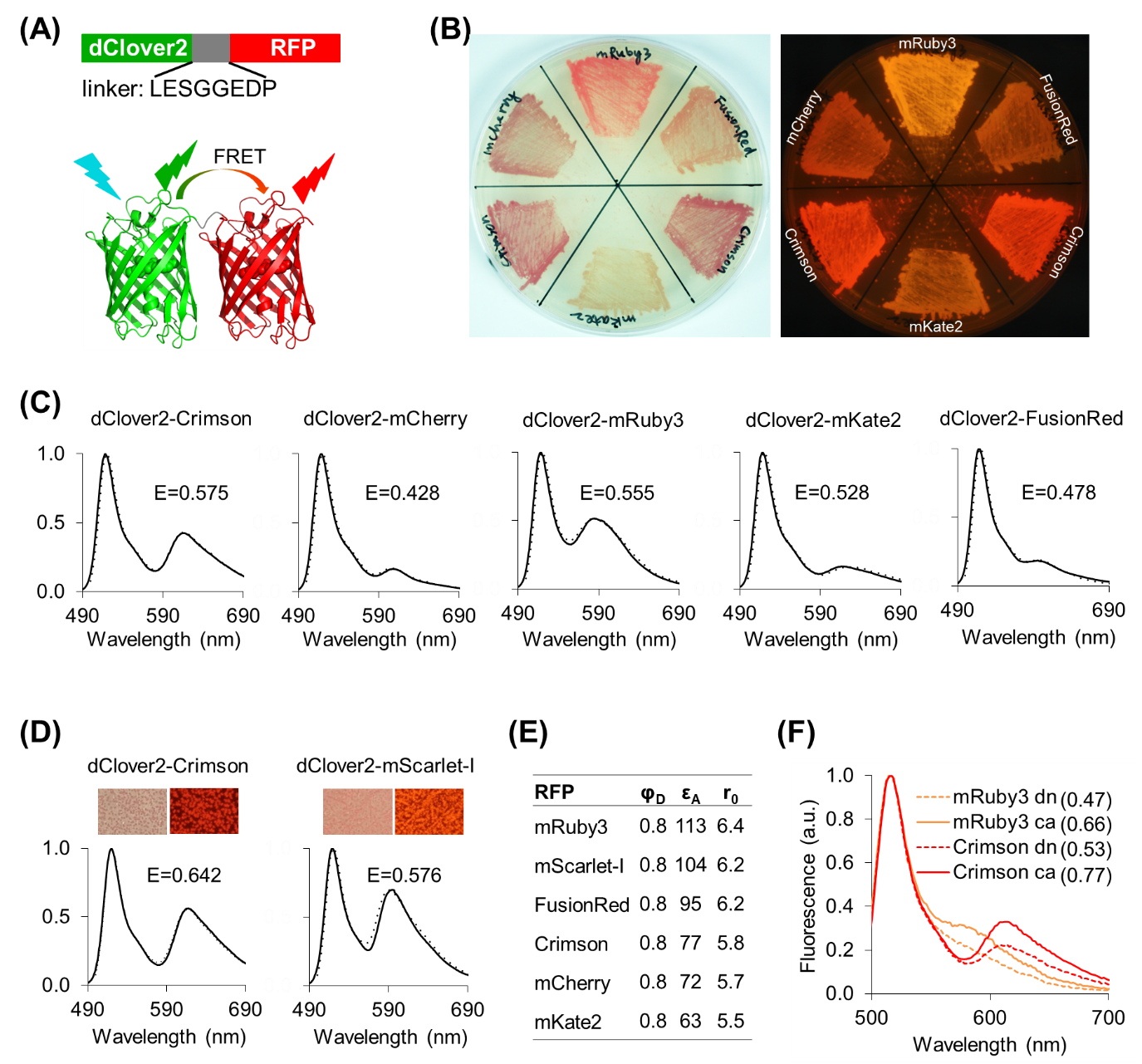


**Supplementary Figure 4. Performance of Crimson as a FRET acceptor. (A)** Schematic diagram of green-red fusion proteins. dClover2-RFP constructs consist of C-terminus truncated dClover2 (no ‘GITHGMDELYK’) and full-length RFPs. In this case, high energy transfer occurs between dClover2 and RFP. **(B)** Bright-field (left) and fluorescence (right) images of patches expressing all fusions except dClover2-mScarlet-I. All patches were incubated at 34°C for 22 h and kept at 4°C for 72 h to ensure all RFPs fully matured. Fluorescence images were acquired with 400- to 500-nm excitation light and a yellow acrylic long-pass filter in a BlueView Transilluminator (Vernier). **(C)** The emission spectra experimentally obtained (solid lines) were fit to linear combinations of emission spectra of dClover2 and RFP (dotted lines). Fluorescence emission spectra were taken on clear lysates with 460 nm excitation. **(D)** Comparison of dClover2-Crimson and dClover2-mScarlet-I in FRET efficiency. All conditions are same as in (b) except that colonies instead of patches were kept at 4°C for 9 days. **(E)** r_0_ values for dClover2-RFP FRE pairs. Φ_D_: quantum yield of donor, ε_A_: extinction coefficient of acceptor in mM^-1^cm^-1^, r_0_: calculated Förster radius assuming random interfluorophore orientation (κ^2^=2/3). **(F)** The emission spectra of intact HeLa cells expressing RhoA sensors based on dClover2-RFP pairs. dn and ca stand for dominant-negative (low FRET) and constitutively active (high FRET) variants of RhoA, respectively. The numbers in brackets are red/green emission ratio, whose changes for dClover2-Crimson and dClover2-mRuby3 are 43.8% and 40.4%, respectively.

**
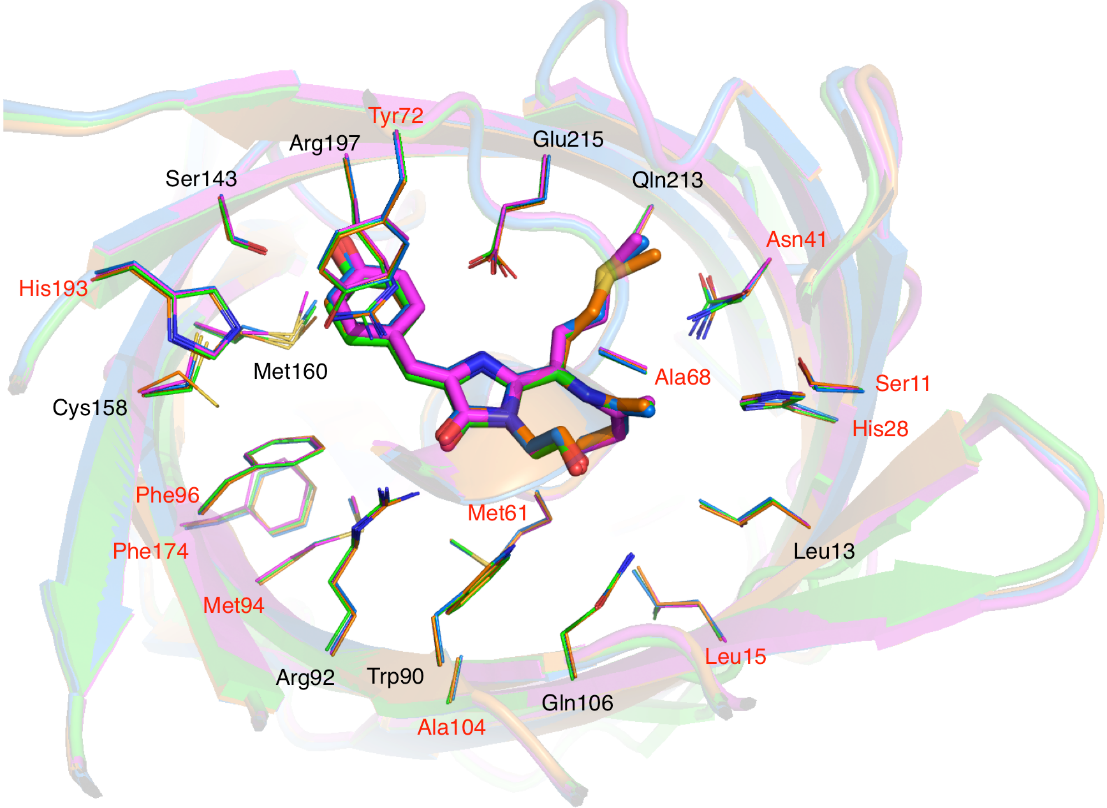
**

**Supplementary Figure 5. Alignment of monomers within the Crimson0.9 crystal unit.** Chromophores and the residues near the chromophore (inner mutations in red) are shown as sticks and lines, respectively. Chains A, B, C, and D are colored in green, blue, megenta and orange, respectively.

**Supplementary Figure 6. dTomato-CAAX forms large rod-like structures in neurons.** dTomato-CAAX fluorescence was imaged in neurons at 15 DIV with a 10× objective (top) or 12 DIV with a 63× objective (lower panels). Arrows indicate rod-like structures. These structures usually protrude from the neuronal membrane, and have linear edges suggestive of a polymerization or crystallization process (lower-right). Scale bar = 50 µm.

**Supplementary Table 1. Summary of mutations identified in each Crimson variant during evolution**

| **Variant** | **Library** | **Rationale** | **Mutations** | **Improvements** |
| --- | --- | --- | --- | --- |
| Crimson0.1 | S28STCDENQKHR | repacking the chromophore | S28H | blue-shifted spectra, less green component, and improved brightness |
|  | G41GASTCDENQKHR |  | G41N |  |
|  | M11X, L13X, S143X, C158X |  | M11S |  |
| Crimson0.2 | I43ILMV, F55FW, C61CS, T94TM, Y96YF | Shuffling of RFP's Allelic Sites with Structural Information. All are inside the barrel | C61S, T94M, Y96F | blue-shifted spectra and improved brightness |
|  | L102LIV, V104VA, T108TS, L115LF |  | L201V |  |
|  | L121FILV, L147LMV, L174LF, T176TS |  | L121V, L174F |  |
| Crimson0.3 | L13MLVSTA, M15ML, C26CIV | Shuffling of Allelic Sites of RFPs with Structural Information. All are inside the barrel | M15L | blue-shifted spectra and improved brightness |
|  | M94MTI, V104VA, V119VA, V121VLIF |  |  |  |
| Crimson0.4 | N71NK, T73TP, Q74QK | The loop 71-74 in FusionRed protein affects protein folding | N71K, T73P | better folding |
|  | G75GD, C158CA |  | Q74K |  |
| Crimson0.5 | H72HY, L138LKR, A142AP, S143ST | Val93 is in the AC dimerization interface and Fhe194 is in the AB dimerization interface | A142P | more monomeric |
|  | V165VDE, Y193YH, F194FYA, V93ST |  | F194Y, V93T |  |
| Crimson0.6 | H10HP, Y14YV, T18TS, N21G, H23HQ | Introducing smaller, hydrophilic amino acids and potential electrostatic interaction and hydrogen bonding | T18S, N21G, H23Q | better folding |
|  | C61CS, K71KN, H72HY, K74KQP, G75GD |  | H72Y |  |
|  | I76IL, F79FY, N118NH, K120KQE, S128SA |  | I76L, F79Y, S128A |  |
|  | P131AP, L138LK, V165VD, N173NK |  |  |  |
|  | K175KE, K185KT, Y193YH, N207ND |  | K185T, Y193H |  |
|  | V218VE |  |  |  |
| Crimson0.7 | C61CSAVTMQL, M94TNQVILMF | Shuffling of Allelic Sites of RFPs with Structural Information. All are inside the barrel | C61M | blue-shifted spectra and improved brightness |
|  | F96FY, V102VCILMF, V104VA |  | V102L, V104A |  |
|  | V121VIL, F174FILMV |  | V121L |  |
| Crismon0.75 | L15LM, L58LIVM, A59ASTV, T60ASTCVIP | Repacking Met61 |  | nothing |
|  | V119ACVILM, L121VILMF, Q213QLM |  |  |  |
| Crimson0.8 | T95TLIVRN, P142AP, K203KEN | Shuffling of Allelic Sites of RFPs with Structural Information. All are outside the barrel | T95V | improved brightness |
|  | T209TM, Y210YFI, R179RKMLI |  | R179K |  |
|  | G153GASTVC |  | G153A |  |
| Crimson0.85 | C172CAVST, P183PT | Repacking Cys172 and optimizing the loop 184-186 |  | nothing |
|  | A184AV, L187LI |  |  |  |
| Crimson0.9 | T68TVPLAS, T89TKRNQ | Shuffling of Allelic Sites of RFPs with Structural Information. | T68A | improved brightness |
|  | G156GASV, I202ILV, Q134QK, F69FY |  | Q134K |  |
|  | K135KRNQCST, K136KRMTED |  | K135Q, K136T |  |
| Crimson0.95 | R220RK, Y221YFH, T209TM, Y210YF | Shuffling of Allelic Sites of RFPs with Structural Information. All are outside the barrel |  | nothing |
|  | E212EV, H214HYR, V216VHR |  |  |  |
| Crimson | H10HPTNR, C114CDEST | mCherry-like | H10R, C114E | more photostable |
|  | K175KV, Y194YNA, L187LV |  | L187V |  |
|  | deletion of the loop 184-186 |  |  |  |

| **Supplementary Table 2. Crimson0.9 X-ray diffraction data collection and refinement statistics** | |  |
| --- | --- | --- |
| Parameter | Value |  |
| Collection wavelength (Å) | 0.9795 |  |
| Space group | P2_1_2_1_2_1_ |  |
| Cell dimensions (Å) | 71.546, 90.046, 129.518 |  |
| Resolution (Å)* | 40.0-2.04 (2.16-2.04) |  |
| Rsym* | 0.108 (0.874) |  |
| I/σ* | 12.12 (2.20) |  |
| Completeness (%)* | 99.1 (95.2) |  |
| Redundancy* | 7.05 (6.90) |  |
| Rwork/Rfree | 0.195/0.231 |  |
| Average B factor | 35 |  |
| RMS deviation, bond lengths (Å) | 0.01 |  |
| RMS deviation, bond angles (°) | 1.18 |  |
| *Statistics for the highest resolution shell are shown in parentheses. | | |
| Data were collected at the Stanford Synchrotron Radiation Laboratory on Beamline 12-2. | | |
| Coordinates were deposited at the Protein Data Bank (PDB) with accession number 6MKP. | | |
